# The relation of eye movements to the occurrence of freezing of gait in Parkinson’s disease

**DOI:** 10.1093/braincomms/fcaf402

**Published:** 2025-10-15

**Authors:** Juan Fernandez-Ruiz, Heidi C Riek, Donald C Brien, Brian C Coe, David A Grimes, Anthony E Lang, Connie Marras, Mario Masellis, Richard H Swartz, Brian Tan, Malcolm A Binns, Stephen R Arnott, the ONDRI Investigators, Sabrina Adamo, Rob Bartha, Courtney Berezuk, Alanna Black, Michael Borrie, Susan Bronskill, Dennis Bulman, Leanne Casaubon, Ben Cornish, Sherif Defrawy, Allison Dilliott, Roger A Dixon, Sali Farhan, Frederico Faria, Julia Fraser, Morris Freedman, Mahdi Ghani, Barry Greenberg, Hassan Haddad, Ayman Hassan, Wendy Hatch, Rob Hegele, Melissa Holmes, Chris Hudson, Mandar Jog, Peter Kleinstiver, Donna Kwan, Elena Leontieva, Brian Levine, Efrem Mandelcorn, Ed Margolin, Bill McIlroy, Manuel Montero-Odasso, David Munoz, Nuwan Nanayakkara, Miracle Ozzoude, Joel Ramirez, Natalie Rashkovan, John Robinson, Ekaterina Rogaeva, Yanina Sarquis Adamson, Christopher Scott, Michael Strong, Sujeevini Sujanthan, Sean Symons, Athena Theyers, Angela Troyer, Karen Van Ooteghem, John Woulfe, Mojdeh Zamyadi, Douglas P Munoz

**Affiliations:** Departamento de Fisiología, Facultad de Medicina, Universidad Nacional Autónoma de México, Ciudad de Mexico CP 04510, México; Instituto de Neuroetología, Universidad Veracruzana, Xalapa, CP 91190 Veracruz, México; Centre for Neuroscience Studies, Queen’s University, Kingston, ON, Canada, K7L 3N6; Centre for Neuroscience Studies, Queen’s University, Kingston, ON, Canada, K7L 3N6; Centre for Neuroscience Studies, Queen’s University, Kingston, ON, Canada, K7L 3N6; Division of Neurology, Department of Medicine, University of Ottawa, Ottawa, ON, Canada, K1Z 8L6; Departmen of Medicine,Ottawa Hospital Research Institute, Ottawa, ON, Canada, K1H 8L6; Brain and Mind Research Institute, University of Ottawa, Ottawa, ON, Canada, K1H 8M5; Edmond J. Safra Program in Parkinson's Disease and the Morton and Gloria Shulman Movement Disorders Clinic, University Health Network, Toronto, ON, Canada, M5T 2S8; Tanz Centre for Research in Neurodegenerative Diseases, University of Toronto, Toronto, ON, Canada, M5S 3H2; Krembil Brain Institute, University Health Network, Toronto, ON, Canada, M5T 1M8; Rossy PSP Centre, University Health Network, Toronto, ON, Canada, M5T 2S8; Edmond J. Safra Program in Parkinson's Disease and the Morton and Gloria Shulman Movement Disorders Clinic, University Health Network, Toronto, ON, Canada, M5T 2S8; Krembil Brain Institute, University Health Network, Toronto, ON, Canada, M5T 1M8; Hurvitz Brain Sciences Program, Sunnybrook Research Institute, University of Toronto, Toronto, ON, Canada, M4N 3M5; Division of Neurology, Department of Medicine, Sunnybrook Health Sciences Centre and University of Toronto, Toronto, ON, Canada, M4N 3M5; Cognitive and Movement Disorders Clinic, Sunnybrook Health Sciences Centre, Toronto, ON, Canada, M4N 3M5; Hurvitz Brain Sciences Program, Sunnybrook Research Institute, University of Toronto, Toronto, ON, Canada, M4N 3M5; Division of Neurology, Department of Medicine, Sunnybrook Health Sciences Centre and University of Toronto, Toronto, ON, Canada, M4N 3M5; Rotman Research Institute, Baycrest Academy for Research and Education, North York, ON, Canada, M6A 1W1; Rotman Research Institute, Baycrest Academy for Research and Education, North York, ON, Canada, M6A 1W1; Dalla Lana School of Public Health, University of Toronto, ON, Canada, M5T 3M7; Indoc Research, Toronto, ON, Canada, M5H 3W4; Centre for Neuroscience Studies, Queen’s University, Kingston, ON, Canada, K7L 3N6; Department of Biomedical and Molecular Sciences, Queen’s University, Kingston, ON, Canada, K7L 3N6

**Keywords:** freezing of gait, Parkinson's disease, anti-saccade, eye movement biomarkers, predictive markers

## Abstract

Freezing of gait is a debilitating motor symptom in Parkinson's disease that significantly increases fall risk and impairs quality of life. The poorly understood pathophysiology of freezing of gait presents challenges for early prediction and therapeutic intervention. This prospective study investigated whether eye movement abnormalities, specifically in the anti-saccade paradigm, could predict freezing of gait onset in Parkinson's disease patients over a two-year follow-up period. We analysed longitudinal data from the Ontario Neurodegenerative Disease Research Initiative, focusing on Parkinson's disease patients without freezing of gait at baseline who underwent comprehensive clinical evaluations and eye movement recordings. Anti-saccade reaction time and error ratio, combined with clinical measures including right upper extremity rigidity, demonstrated significant predictive value for freezing of gait development within two years. These findings suggest that eye movement deficits and upper limb rigidity emerge years before freezing of gait onset, indicating a prodromal phase in freezing of gait pathogenesis. The predictive relationship between these measures supports the hypothesis of shared neural substrates, potentially involving the mesencephalic locomotor region, in the development of both oculomotor dysfunction and gait freezing episodes.

## Teaser

Fernandez-Ruiz *et al*. report that specific eye movement abnormalities, particularly anti-saccade error patterns, can predict the development of freezing of gait in Parkinson's disease patients within two years. These oculomotor deficits serve as early prodromal markers, offering potential for timely clinical interventions before gait freezing symptoms emerge.

## Graphical Abstract

**Graphical Abstract:**
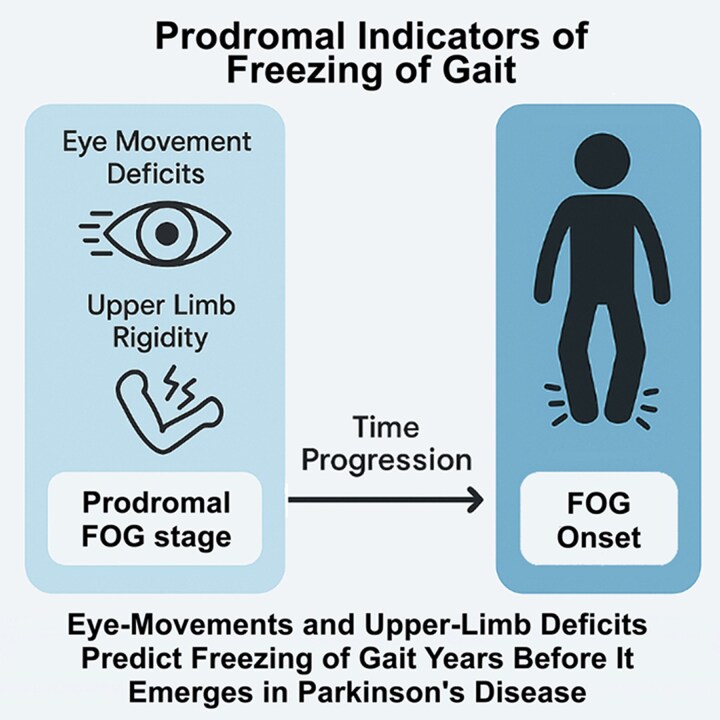

## Introduction

Parkinson's disease (PD) is a progressive neurodegenerative disorder marked by motor symptoms such as bradykinesia, rigidity, tremor, and postural instability.^1^ Freezing of Gait (FOG), a transient inability to walk despite the intention to do so, is particularly debilitating, often causing falls and reducing quality of life.^1^ The pathophysiology of FOG remains unclear, complicating its prediction and management.^2^

Eye movement abnormalities, including disturbances of saccades and smooth pursuit, have been linked to motor and non-motor symptoms in PD.^3,4^ Neural circuits controlling eye movements overlap with those involved in gait and balance,^5,6^ suggesting that pathophysiological changes in these circuits may impact oculomotor performance, gait, and balance, including the emergence of FOG.^7-10^

This study examined whether early alterations in saccadic eye movements could predict FOG in PD. Using data from the Ontario Neurodegenerative Disease Research Initiative (ONDRI), we analysed eye movement and clinical assessments over two years to identify early predictors of FOG.^11-13^

## Materials and methods

### Participants

One hundred patients with idiopathic PD,^14^ Hoehn & Yahr scores of 1–3, and normal or corrected-to-normal vision completed baseline assessments, including the Freezing of Gait Questionnaire (FOG-Q)^15^ and oculomotor evaluations ON medication. At the two-year follow-up, 66 participants remained active in the study, including 21 who were FOG-free at baseline. These 21 patients were divided into two groups based on their FOG status after two years: those who remained FOG-free (*n*-FOG, *n* = 10) and those who developed FOG (y-FOG, *n* = 11) (see supplemental materials for detailed participant descriptions).

Ethical approval was obtained, and participants provided written consent according to the Declaration of Helsinki. Because the ONDRI battery required several hours, participants remained ON their usual levodopa regimen. Sustained medication withdrawal was considered impractical and uncomfortable for the patients. Approval for experimental procedures was obtained from the Queen’s University Health Sciences and Affiliated Teaching Hospitals Research Ethics Board and research ethics committees at all participating ONDRI recruitment sites.

### Clinical assessments

Each participant underwent clinical evaluations, including the Montreal Cognitive Assessment (MoCA),^16^ Hoehn & Yahr scale (HY),^17^ and Movement Disorder Society-Unified Parkinson's Disease Rating Scale (MDS-UPDRS) (total and Part III scores).^18^ Assessments were conducted at baseline and two-year follow-up.^19^ FOG frequency and severity were assessed using the six-item FOG-Q, scored on a 5-point scale, with higher scores indicating more severe FOG.^15^

### Eye movement evaluation

The interleaved pro/anti-saccade task (IPAST) was used to evaluate all participants, using an infrared video-based eye tracker with a sampling rate of 500 Hz..^13,19,20^ For detailed methods, data preprocessing and saccade classification, see supplementary materials.^20^

We calculated saccade reaction time (SRT), subdivided into express-latency (90–139 ms) and regular-latency (140–800 ms) for pro- and anti-saccades.^13,21^ Percentages of express- and regular-latency direction errors and fixation breaks were computed for pro- and anti-saccade trials. Additionally, the mean amplitude of correct pro- and anti-saccades was calculated, providing a comprehensive summary of oculomotor behaviours across all viable responses.

### Statistical analysis

Statistical tests compared the *n*-FOG and y-FOG groups. Independent samples t-tests assessed differences in age, disease duration, HY scale scores, MDS-UPDRS, levodopa equivalent daily doses (LEDD),^22^ and MoCA scores, while a chi-square test of independence evaluated sex distribution differences. For preprocessing, a two-step approach optimized feature scaling of eye-movement and clinical data. First, Winsorization adjusted extreme values beyond the 1st and 99th percentiles to these thresholds, reducing outlier influence while preserving data integrity.^23^ Next, min-max standardization scaled data linearly between 0 and 1 using the formula *x*_normalized_={*x* – *x*_min_/*x*_max_ – *x*_min_}​​, ensuring proportional feature contributions. This preprocessing framework improved data reliability and analysis, supporting algorithms sensitive to input magnitudes and providing a strong foundation for comparing clinical and oculomotor features across groups.

Longitudinal analyses of demographics, motor evaluation, cognitive screening, and FOG severity were conducted using mixed-effects modelling, suitable for repeated measures and unbalanced datasets. Group comparisons were performed using MANCOVA to assess the effect of group (*n*-FOG versus y-FOG) on baseline eye-movement and other variables, controlling for age and disease duration. Post hoc Tukey's HSD tests explored specific differences after significant MANCOVA results, controlling the family-wise error rate and Type I errors across multiple comparisons.^24^

Receiver Operating Characteristic (ROC) curve analysis assessed the classification ability of variables with significant group differences to predict FOG. ROC curves plotted true positive rate (TPR) against false positive rate (FPR), while Youden's Index (TPR—FPR) identified optimal thresholds maximizing sensitivity and specificity. Higher Youden's Index values indicated better discriminatory ability. Area Under the Curve (AUC) quantified overall performance, with values closer to 1 reflecting superior accuracy, ensuring robust evaluation of each variable's predictive capability.

## Results

### Demographic and clinical screening results.

There were no significant differences between the *n*-FOG and y-FOG groups at baseline in terms of age, disease duration or sex distribution. Clinical evaluations conducted during the first session also indicated no significant differences in the scores of the HY scale, the MDS-UPDRS, LEDD, or MoCA (see supplementary results for details).

Longitudinal analyses were performed on the HY scale, MDS-UPDRS, LEDD, MoCA and FOG-Q score data. Mixed-effects model analyses were conducted to assess the effects of group (*n*-FOG and y-FOG) and time (baseline and follow-up) (Supplementary Table I), taking into account the individual change over time. In summary, the groups were homogeneous at the beginning. However, for MoCA and FOG-Q, there was a significant interaction effect between group and time, suggesting that the y-FOG group experienced a larger longitudinal decline in these scores compared to the *n*-FOG group (see supplementary results for details).

### Eye movement results

The main aim of this study was to explore whether eye movement variables recorded at baseline differed between individuals who would or would not develop FOG two years later.^13^

### Pro-saccades (PS)

**Figure 1:**
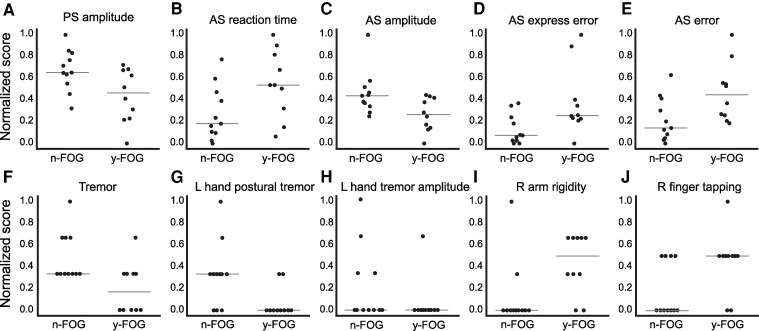
**Normalized metrics that differed between groups at the first visit.** Group effects (*n*-FOG (*n* = 10) and y-FOG (*n* = 11)) are from the primary models (MANCOVA with variable-level follow-up tests, adjusted for covariates as specified in Methods). Panels display individual data points where each dot represents one participant; central horizontal lines indicate medians. A-E, Eye-movement variables: (**A)** PS amplitude (*P* = 0.02, *η*² = 0.12); (**B)** AS reaction time (*P* = 0.03, η² = 0.2); (**C)** AS amplitude (*P* = 0.02, *η*² = 0.24); (**D**) AS express error (*P* = 0.02, *η*² = 0.23); (**E**) AS error (*P* = 0.02, *η*² = 0.23). F-J, Motor variables: (**F**) tremor (*P* = 0.01, *η*² = 0.29); (**G**) left hand postural tremor (*P* = 0.01, *η*² = 0.25); (**H**) left hand tremor amplitude (*P* = 0.01, *η*² = 0.35); (**I**) right arm rigidity (*P* = 0.02, *η*² = 0.23); (**J**) right finger tapping (*P* = 0.03, *η*² = 0.21). PS: pro-saccade; AS: antisaccade.

MANCOVA revealed significant group effects on PS variables, Wilks’ λ = 0.44, *F*(5, 13) = 3.21, *P* = 0.04, *η*² = 0.55, with no significant effects of age or disease duration. Tukey's HSD showed significant group differences in PS amplitude (*P* = 0.02, *η*² = 0.12), with larger amplitudes in the *n*-FOG group (Fig.1). No differences were found for PS SRT, errors, or fixation breaks. Longitudinal analysis showed significant group differences in PS amplitude (*P* = 0.01, *η*² = 1.16), but no time (*P* = 0.09, *η*² = 0.09), or interaction effects (*P* = 0.79, *η*² = 0.0).

### Anti-saccades (AS)

MANCOVA revealed significant group effects, Wilks’ λ = 0.30, F(5, 13) = 5.99, *P* = 0.00, *η*² = 0.69. Tukey's HSD showed group differences in anti-saccade SRT (*P* = 0.03, *η*² = 0.2), amplitude (*P* = 0.02, *η*² = 0.24), express-latency error ratio (*P* = 0.02, η² = 0.23), and regular-latency error ratio (*P* = 0.02, *η*² = 0.23) (Fig.1 A), but not fixation breaks. Mixed-effects analysis showed significant group differences and time effects only for anti-saccade SRT (*P* = 0.01, *η*² = 1.06 and *P* = 0.01, *η*² = 0.26), and regular-latency error ratio (*P* = 0.01, *η*² = 1.18 and *P* = 0.03, η² = 0.23), but no interactions (*P* = 0.8, *η*² = 0.01 and *P* = 0.58, *η*² = 0.03 respectively). Results suggest significant oculomotor differences existed before FOG onset, without larger longitudinal declines in the y-FOG group.

### MDS-UPDRS

MANCOVA revealed significant group effects on motor-related clinical variables, Wilks’ λ = 0.19, *F*(10, 8) = 3.39, *P* = 0.048, η² = 0.81, with no significant effects of age or disease duration. Tukey's HSD showed group differences in tremor (*P* = 0.01, *η*² = 0.29), postural tremor left hand LH (*P* = 0.01, *η*² = 0.25), resting tremor amplitude LH (*P* = 0.01, *η*² = 0.35), right arm rigidity (*P* = 0.02, η² = 0.23), and RH finger tapping (*P* = 0.03, *η*² = 0.21) (Fig.1 B). No differences were found for right-hand (RH) postural tremor, RH amplitude tremor, LH rigidity, or LH finger tapping, though neck rigidity had a *P* = 0.052.

### ROC analyses

**Figure 2:**
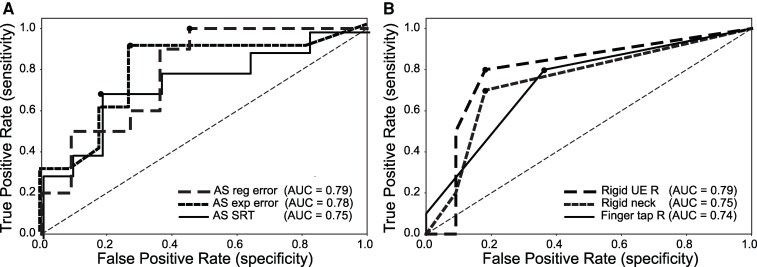
**Receiver operating characteristic (ROC) curves for the only variables predicting freezing of gate (FOG).** The dots on each curve mark the optimal cut-off points as determined by Youden's Index. (**A**) Eye-movement variables. (**B**) Clinical variables. AUC: area under the curve. Left panel lines are shown jittered for clarity purposes.

ROC analyses evaluated the classification ability of variables to predict FOG development (Fig. 2, Supplementary Table 2). AS regular-latency error ratio and AS express-latency error ratio showed the highest diagnostic ability (AUC: 0.79, 0.78), with high sensitivity (1.00, 0.90) and reasonable specificity (0.55, 0.73), supported by Youden's Index (0.55, 0.63). Right upper extremity rigidity also performed well (AUC: 0.79, sensitivity: 0.80, specificity: 0.82, Youden's Index: 0.62). Moderate predictors included AS SRT, neck rigidity (AUC: 0.75, Youden's Index: 0.52), and right finger tapping (AUC: 0.74, Youden's Index: 0.44). Poor predictors included PS amplitude and tremor variables (AUC: 0.20, Youden's Index: 0.0).

## Discussion

This study investigated whether eye movement variables predict the development of FOG in PD patients over two years. We found that AS variables (regular-latency error ratio, express-latency error ratio) and clinical measures (e.g. right upper extremity rigidity) had strong classification ability prior to FOG development. These results highlight the potential for including eye movement assessments with clinical evaluations for early prediction and intervention (as effective treatments become available) for FOG in PD patients.

Our findings are supported by prior studies showing impaired saccadic latencies,^7,8^ and anti-saccade errors^9^ in PD patients with FOG. Our results further identified useful AS SRT thresholds, highlighting its utility as a marker.

The neurological relationship between eye movement deficits and FOG in PD is underpinned by several key neural mechanisms. Disruptions in fronto-parietal networks involved in cognitive control and inhibitory processes affect anti-saccade performance and automatic and voluntary saccades.^9,25,26^ Additionally, basal ganglia dysfunction affects the initiation and execution of both eye and limb movements, contributing to the motor disturbances seen in FOG.^8^ Furthermore, prolonged saccade latency correlates with more severe motor symptoms or motor asymmetries in PD patients with FOG.^10,27^ Finally, gait and eye movement control are both affected by altered connectivity within the mesencephalic locomotor region (MLR), including the pedunculopontine nucleus.^5,7,28,29^

Limitations: Testing rigidity and tremor ON medication may blunt symptom severity and limit sensitivity, yet these scores still predicted later FOG. The modest sample size relative to multiple predictors tested raises potential concerns about overfitting; replication in larger cohorts will be important to confirm these predictive relationships.

In conclusion, our findings demonstrate that oculomotor deficits (anti-saccade error ratio and reaction time) combined with lateralized motor signs (right upper extremity rigidity and finger tapping decrements) serve as strong prodromal indicators of FOG development in PD.

## Supplementary Material

fcaf402_Supplementary_Data

## Data Availability

All data collected during the ONDRI foundational study, including all data supporting the findings of this study, are available on request from the Ontario Brain Institute (OBI) (details at https://www.braincode.ca/). The automated analysis pipeline information can be found in Coe et.al 2024 (https://www.mdpi.com/2411-5150/8/1/14).

## Appendix

**ONDRI Investigators:** Sabrina Adamo, Rob Bartha, Courtney Berezuk, Alanna Black, Michael Borrie, Susan Bronskill, Dennis Bulman, Leanne Casaubon, Ben Cornish, Sherif Defrawy, Allison Dilliott, Roger A. Dixon, Sali Farhan, Frederico Faria, Julia Fraser, Morris Freedman, Mahdi Ghani, Barry Greenberg, Hassan Haddad, Ayman Hassan, Wendy Hatch, Rob Hegele, Melissa Holmes, Chris Hudson, Mandar Jog, Peter Kleinstiver, Donna Kwan, Elena Leontieva, Brian Levine, Efrem Mandelcorn, Ed Margolin, Bill McIlroy, Manuel Montero-Odasso, David Munoz, Nuwan Nanayakkara, Miracle Ozzoude, Joel Ramirez, Natalie Rashkovan, John Robinson, Ekaterina Rogaeva, Yanina Sarquis Adamson, Christopher Scott, Michael Strong, Sujeevini Sujanthan, Sean Symons, Athena Theyers, Angela Troyer, Karen Van Ooteghem, John Woulfe and Mojdeh Zamyadi.
