# Supplementary material for: The relation of eye movements to the occurrence of freezing of gait in Parkinson’s disease
Source: Brain Commun. 2025 Oct 15;7(5):fcaf402. doi: 10.1093/braincomms/fcaf402 (PMC12569761; doi:10.1093/braincomms/fcaf402)
Supplement: fcaf402_Supplementary_Data [file fcaf402_supplementary_data.pdf]

Supplementary Material for “The relation of eye movements to the occurrence of freezing of gait in Parkinson's disease”.

Participants

We recruited individuals diagnosed with Parkinson's disease from ONDRI [1–3]. ONDRI is a comprehensive research program designed to enhance the understanding of neurodegenerative diseases [1]. ONDRI's multi-site, prospective cohort study provided us with extensive data, including clinical and eye movement assessments [3]. From the ONDRI platform, we selected all patients with PD who did not exhibit FOG at baseline and then completed the same assessments two years later. This design allowed exploring whether specific eye-movement parameters could serve as early predictors of FOG to facilitate timely intervention and improve patient outcomes. Approval for experimental procedures was obtained from the Queen’s University Health Sciences and Affiliated Teaching Hospitals Research Ethics Board and research ethics committees at all participating ONDRI recruitment sites. Written consent was obtained from each PD participant. At baseline, participants with PD were 57–80 years of age, diagnosed with idiopathic PD based on the United Kingdom Parkinson's Disease Society Brain Bank criteria within the last 3–8 years [4], and had a Hoehn & Yahr score of 1-3. Participants with PD had normal or corrected-to-normal vision in at least one eye, and were on medication as required (Table I).

At the initial visit, one hundred patients underwent comprehensive assessments, completed the Freezing of Gait Questionnaire (FOG-Q) [5], and oculomotor evaluations. At two-year follow-up, sixty-six of the original cohort remained active in the study. Of these, twenty-one had initially reported no freezing of gait at baseline (FOG-Q score of zero). Therefore, the analysis ultimately focused on these participants that were then divided into two groups: ten subjects who did not develop FOG (n-FOG), and eleven subjects who developed FOG over a two-year period (y-FOG).

Clinical assessments

PD clinical evaluation.

Each participant with Parkinson's Disease (PD) underwent a comprehensive neurological examination[1,3]. For this analysis, we utilized the Montreal Cognitive Assessment (MoCA)[6] Hoehn & Yahr scale (HY) [7], and Movement Disorder Society-Unified Parkinson's Disease Rating Scale (MDS-UPDRS) (total score of all four sections and Part III – Motor Examination)[8]. These assessments were conducted at the baseline visit and during follow-up at year 2 (Supplementary Table I)[9].

Freezing of gait evaluation.

FOG was evaluated with the FOG-Q, which assesses the severity and frequency of FOG in patients with PD. It consists of six items, each scored on a 5-point interval scale (ranging from 0 to 4). Higher scores indicate more severe FOG[5].

Eye movement evaluation.

Task and eye-movement recordings.

The interleaved pro/anti-saccade task (IPAST) was used to evaluate all participants [9–11]. Participants sat approximately 60 cm away from a 17-inch computer monitor with 1280 x 1024-pixel resolution. Gaze position was tracked monocularly using an infrared video-based eye tracker (EyeLink 1000 Plus, SR Research Ltd., Ottawa, ON, Canada) with sampling rate of 500 Hz. Calibration was usually performed using a nine-point array, or a five-point array if the nine-point array was unsuccessful. Each IPAST trial commenced with a 1000 ms inter-trial interval (ITI), featuring a blank black background (0.1 cd/m2). Following this, a central fixation point (FIX; 0.5° diameter dot, 44 cd/m2) appeared for 1000 ms. The color of the FIX indicated the type of trial: green for a pro-saccade trial (PRO) and red for an anti-saccade trial (ANTI). After the FIX disappeared, the screen returned to a blank state for 200 ms during the gap period (GAP). Subsequently, a peripheral stimulus (STIM; 0.5° diameter dot; gray, 62 cd/m2) emerged 10° horizontally to the left or right of the original FIX position. During PRO trials, participants were directed to quickly move their gaze to the STIM upon its appearance. Conversely, in ANTI trials, participants were instructed to look in the opposite direction. Direction errors occurred when the first saccade after STIM onset was contrary to the trial's instructions. The conditions of the task (PRO or ANTI) and the STIM locations (left or right) were mixed pseudo-randomly and appeared with equal frequency. The IPAST experiment lasted about 20 minutes in total divided into two blocks each comprising 120 trials. This task allowed us to collect automatic and volitional saccades [11].

Calculation of oculomotor measures.

Data preprocessing and saccade classification were performed by custom in-house scripts written in MATLAB (The MathWorks Inc., Natick, MA, USA) [10], which removed from analysis trials with lost tracking and abnormal behaviour (e.g., no saccade, saccades to random locations, failure to fixate centrally during the fixation period, or saccade reaction time >800 ms). We categorized behaviour in the remaining trials as follows:

Saccade reaction time (SRT) refers to the time between STIM appearance and first saccade initiation. We considered viable any saccade responses with SRT 90-800 ms, due to the 90 ms afferent delay for visually-triggered saccades, and further subdivided into express-latency (SRT 90-139 ms) and regular-latency saccades (SRT 140-800 ms), which result from different underlying processes and are differentially modulated by pathology [11,12] . We computed the median SRT of viable responses for both pro-saccades and anti-saccades separately. We also calculated the percentages of express-latency and regular-latency pro- and anti-saccade direction errors relative to all viable pro-saccade and anti-saccade responses respectively.

Fixation breaks occur when participants look away from FIX and do not return gaze to FIX before its disappearance [10]. We computed the percentage of fixation breaks, relative to all trials, in pro- and anti-saccade trials separately.

Finally, we calculated the mean amplitude of viable correct pro- and anti-saccades.

Statistical analysis.

The analyses of demographic and clinical results involved several statistical tests to compare the n-FOG and y-FOG groups. Independent samples t-tests were conducted to assess differences in age, disease duration, HY scale scores, UPDRS, levodopa equivalent doses (LEDD) [13], and MoCA scores. Additionally, a Chi-Square Test of Independence was conducted to assess differences in sex distribution between the groups.

Data normalization. For the subsequent analyses, a comprehensive two-step preprocessing of the dataset was utilized to optimize feature scaling of the eye movement and clinical data. Initially, the data was Winsorized to mitigate the influence of outliers, where extreme values beyond the 1st and 99th percentiles were adjusted to these percentile thresholds, thus preserving data integrity while reducing the impact of anomalies [14]. Subsequently, the dataset underwent min-max normalization, a technique that linearly scales the data values between 0 and 1 using the formula $x_{normalized}=\{x - x_{min}/x_{max} - x_{min}\}$. This method ensures that each feature contributes proportionately to the outcomes, making it particularly beneficial for algorithms sensitive to input magnitudes. Together, Winsorization and min-max normalization provide a robust framework for data preparation, enhancing the reliability and effectiveness of subsequent analyses.

Longitudinal analyses of demographics, motor evaluation, cognitive screening, and FOG severity were conducted using mixed-effects modeling, which can handle unbalanced datasets and is suitable for repeated measures data, while providing estimates for the effects of time, group, and their interaction.

Group comparisons. Multivariate analyses of covariance (MANCOVA) were conducted to determine the effect of group (n-FOG vs. y-FOG) on the baseline performance scores of the different eye movement variables, as well as the other variables included in the study while controlling for age and disease duration. Subsequent post hoc analyses using Tukey's HSD tests were used to further explore specific differences after significant MANCOVA results. Note that Tukey's HSD test is designed to handle the family-wise error rate when performing multiple pairwise comparisons after an ANOVA, thus controlling Type I errors across multiple tests so no further correction for multiple comparisons is necessary [15].

The diagnostic ability of each variable that showed significant differences between groups was assessed. This was done using Receiver Operating Characteristic (ROC) curve analysis to predict the development of FOG. This involved plotting the true positive rate (TPR) against the false positive rate (FPR) at various thresholds, providing a visual representation of a variable's performance across different threshold levels. To further evaluate the effectiveness of each variable, Youden's Index was calculated for each threshold. This index, defined as the difference between TPR and FPR, reflects the test's discriminatory ability, with higher values indicating better performance. The optimal cut-off point for each variable was determined by finding the threshold that maximized Youden's Index, ensuring the best balance between sensitivity and specificity. Additionally, the Area Under the Curve (AUC) was calculated for each ROC curve to quantify the overall diagnostic ability, with values closer to 1 indicating superior performance.

Results.

Demographic and clinical screening results.

There were no significant differences between the n-FOG and y-FOG groups at baseline in terms of age ($t=0.65, p=0.51$) or disease duration ($t=-0.02, p=0.97$). Furthermore, there were no differences in sex distribution between the groups ($\chi 2$ (1, N=21) =0.00, p=0.99). Clinical evaluations conducted during the first session also indicated no significant differences in the scores of the HY scale ($t=0.1, p=0.92$), the MDS-UPDRS part III ($t=0.81, p=0.42$), LEDD ($t=0.28, p=0.78$), or MoCA ($t=0.21, p=0.83$).

Longitudinal analyses.

Longitudinal analyses were performed on the HY scale, UPDRS, LEDD, MoCA and FOG-Q score data. Mixed-effects model analyses were conducted to assess the effects of group (n-FOG and y-FOG) and time (baseline and follow-up) (Table I).

For the HY scale, there was no significant main effect of group, $b=-0.02b=-0.02$, SE=0.16, $z=-0.11$, $p=0.909$, $\eta 2=0.00$; however there was a significant effect of time, b = 0.27, SE = 0.12, z = 2.25, p = 0.025, $\eta^2 = 0.56$, but not significant interaction between group and time, b = -0.17, SE = 0.18, z = -0.98, p = 0.326, $\eta^2 = 0.22$.

For UPDRS, there was no significant effect of group, b = -4.59, SE = 6.58, z = -0.70, p = 0.485, $\eta^2 = 0.09$, nor of time, b = 6.09, SE = 4.18, z = 1.46, p = 0.145, $\eta^2 = 0.16$, and no significant interaction between group and time, b = 7.31, SE = 6.06, z = 1.21, p = 0.228, $\eta^2 = 0.24$.

For LEDD, there was no significant main effect of group, b = -24.89, SE = 112.37, z = -0.22, p = 0.825, $\eta^2 = 0.01$, nor time, b = 97.73, SE = 91.37, z = 1.07, p = 0.285, $\eta^2 = 0.14$, or interaction between group and time b = 20.27, SE = 132.41, z = 0.15, p = 0.878, $\eta^2 = 0.01$.

For MoCA, there was no main effect of group b = -0.25, SE = 1.23, z = -0.21, p = 0.837, $\eta^2 = 0.01$, no main effect of time, b = 1.18, SE = 0.86, z = 1.38, p = 0.169, $\eta^2 = 0.18$, but significant interaction between group and time, b = -2.78, SE = 1.24, z = -2.24, p = 0.025, $\eta^2 = 0.97$.

Finally, for FOG-Q scores, the mixed-effect model showed that the main effect of group was not significant, b = 0.00, SE = 0.87, z = 0.00, p = 1.000, $\eta^2 = 0.00$, nor the main effect of time, b = -0.00, SE = 0.85, z = -0.00, p = 1.000, $\eta^2 = 0.00$. However, there was a significant interaction between group and time, b = 3.60, SE = 1.23, z = 2.93, p = 0.003, $\eta^2 = 3.27$.

Supplementary Table I. Demographic, motor evaluation, cognitive screening, and FOG (Freezing of Gait) information. Demographic data includes participant numbers, female/male ratios, mean age, and disease duration. Motor evaluation included the HY (Hoehn and Yahr) scale, MDS-UPDRS part III (International Parkinson and Movement Disorder Society's revision of the Unified Parkinson's Disease Rating Scale) scores, and Levodopa equivalent daily doses (LEDD). Cognitive screening was performed using the MoCA (Montreal Cognitive Assessment) scores. FOG was assessed using FOG-Q (Freezing of Gait Questionnaire) scores. Participants were

categorized into n-FOG (without freezing of gait) and y-FOG (with freezing of gait) groups based on the absence or presence of FOG at 2-year follow-up. The table shows the mean and standard deviations.

**Demographic data**	**Baseline**		**2 yr. follow-up**				
	**n-FOG**	**y-FOG**	**n-FOG**	**y-FOG**	**group**	**time**	**Interaction**
Participant n	11	10	11	10			
Female/male	3/8	2/8	3/8	2/8			
Age (years)	69.4 (5.4)	67.9 (5.1)	71.4 (5.4)	69.9 (5.1)			
Disease duration (years)	6.27 (2.5)	6.3 (2.1)	8.27 (2.5)	8.3 (2.1)			
**Motor evaluation**							
HY scale	1.8 (.4)	1.8 (.42)	2(.3)	1.9(.3)	p=0.9	p=.02	p=0.3
MDS-UPDRS Part III	33 (14.2)	28.5 (11.3)	39.1 (16)	41.9 (17.8)	p=0.4	p=0.1	p=0.2
LEDD	482 (208)	457 (194)	580 (283)	575 (322)	p=0.8	p=0.2	p=0.8
**Neuropsychological test**							
MoCA	26.4 (2.58)	26.2 (2.8)	27.6 (2)	24.6 (3.6)	p=0.8	p=0.1	p =.02
**FOG**							
FOG-Q score	0	0	0	3.6 (4)	p=1.0	p=1.0	p <.01

Supplementary Table 2. ROC analyses of the variables that showed differences between n-FOG and y-FOG groups ranked by AUC**.**

**Variable**	**AUC**	**Optimal Threshold**	**Sensitivity**	**Specificity**	**Youden's Index**
AS error ratio	0.79	0.19	1	0.55	0.55
Right upper extremity rigidity	0.79	0.33	0.8	0.82	0.62
AS express error ratio	0.78	0.21	0.9	0.73	0.63
AS reaction time	0.75	0.51	0.7	0.82	0.52
Neck rigidity	0.75	0.5	0.7	0.82	0.52
Right finger tapping	0.74	0.5	0.8	0.64	0.44
PS amplitude	0.23	inf	0	1	0
Left hand postural tremor	0.22	inf	0	1	0
Left upper extremity resting tremor amplitude	0.2	inf	0	1	0
AS amplitude	0.2	inf	0	1	0
Tremor	0.2	inf	0	1	0

Discussion of lateralization. The lateralized pattern we observed is intriguing and may reflect several underlying mechanisms. However, lateralization in FOG remains complex and incompletely understood. Right-sided motor symptoms in Parkinson's disease typically correspond to greater dopaminergic dysfunction in the left hemisphere, which plays a critical role in motor sequencing, executive function, and cognitive control—functions essential for navigating complex gait tasks that provoke freezing of gait (FOG)[16]. Thus, left-hemispheric dysfunction may disproportionately impair neural circuits responsible for adaptive locomotion, making right-sided rigidity and finger tapping deficits more predictive of FOG development. Furthermore, it has also been found that patients with right-sided motor symptoms exhibited worse gait parameters—including shorter step length and increased step time variability compared to those with left-sided symptoms, potentially reflecting greater left-hemisphere involvement in PD-FOG pathophysiology [17]. This aligns with our finding that right-sided motor deficits are more predictive of future FOG emergence.

We acknowledge these interpretations are speculative, particularly given our limited sample size. Future studies with larger cohorts and neuroimaging data should explore hemispheric contributions to FOG more directly to clarify these mechanistic relationships

Medication Review Report: n-FOG vs. y-FOG Groups

1. Overview

- Total Subjects: 21
- Total Medications: 103
- Average Medications per Subject: 4.9
- Most Common Medication: Aspirin (38.1%)

2. Group Comparison

Metric	n-FOG (n=11)	y-FOG (n=10)
Avg. Medications	4.1	5.8
Range	1–11	1–18
Median	3.0	4.0
Aspirin Use	27.3%	50.0%
Cardiovascular Meds	45.5%	70.0%
Mental Health/Sleep Meds	54.5%	20.0%
Supplement Use	18.2%	40.0%

3. Key Findings

Medication Burden

- y-FOG subjects take 41% more medications on average.
- Greater variability in y-FOG (notably Subject 17 with 18 medications, 13 of which are supplements).
- Polypharmacy (≥7 meds):
  - y-FOG: 3/10 subjects
  - n-FOG: 2/11 subjects

Aspirin Usage

- n-FOG: Used for pain and general blood thinning.
- y-FOG: Primarily for cardiovascular protection.

Therapeutic Focus

- n-FOG: Higher use of neuropsychiatric medications (e.g., lorazepam).
- y-FOG: More cardiovascular agents (e.g., atorvastatin, tamsulosin) and supplements (e.g., Vitamin B12, Omega 3).

4. Clinical Implications

- y-FOG Group:
  - Higher cardiovascular medication burden
  - Aspirin used primarily for cardioprotection
  - More age-related/metabolic comorbidities
  - Higher supplement usage patterns

- n-FOG Group:
  - Higher mental health/sleep medication usage
  - Aspirin used more for pain management
  - More neuropsychiatric comorbidities
  - Lower overall medication burden

References

1. Farhan SMK, Bartha R, Black SE, et al. The Ontario Neurodegenerative Disease Research Initiative (ONDRI). *Can J Neurol Sci J Can Sci Neurol*. 2017;44(2):196-202. doi:10.1017/cjn.2016.415
2. Riek HC, Brien DC, Coe BC, et al. Cognitive correlates of antisaccade behaviour across multiple neurodegenerative diseases. *Brain Commun*. 2023;5(2):fcad049. doi:10.1093/braincomms/fcad049
3. Sunderland KM, Beaton D, Arnott SR, et al. Characteristics of the Ontario neurodegenerative disease research initiative cohort. *Alzheimers Dement*. 2023;19(1):226-243.
4. Hughes AJ, Daniel SE, Kilford L, Lees AJ. Accuracy of clinical diagnosis of idiopathic Parkinson's disease: a clinico-pathological study of 100 cases. *J Neurol Neurosurg Psychiatry*. 1992;55(3):181-184.
5. Giladi N, Tal J, Azulay T, et al. Validation of the freezing of gait questionnaire in patients with Parkinson's disease. *Mov Disord Off J Mov Disord Soc*. 2009;24(5):655-661.
6. Nasreddine ZS, Phillips NA, Bédirian V, et al. The Montreal Cognitive Assessment, MoCA: a brief screening tool for mild cognitive impairment. *J Am Geriatr Soc*. 2005;53(4):695-699.
7. Hoehn MM, Yahr MD. Parkinsonism: onset, progression, and mortality. *Neurology*. 1967;17(5):427-427.
8. Goetz CG, Tilley BC, Shaftman SR, et al. Movement Disorder Society-sponsored revision of the Unified Parkinson's Disease Rating Scale (MDS-UPDRS): scale presentation and clinimetric testing results. *Mov Disord Off J Mov Disord Soc*. 2008;23(15):2129-2170.
9. Brien DC, Riek HC, Yep R, et al. Classification and staging of Parkinson's disease using video-based eye tracking. *Parkinsonism Relat Disord*. 2023;110:105316. doi:https://doi.org/10.1016/j.parkreldis.2023.105316
10. Coe BC, Huang J, Brien DC, White BJ, Yep R, Munoz DP. Automated analysis pipeline for extracting saccade, pupil, and blink parameters using video-based eye tracking. *Vision*. 2024;8(1):14.
11. Riek HC, Brien DC, Coe BC, et al. Cognitive correlates of antisaccade behaviour across multiple neurodegenerative diseases. *Brain Commun*. 2023;5(2):fcad049.
12. Coe BC, Munoz DP. Mechanisms of saccade suppression revealed in the anti-saccade task. *Philos Trans R Soc B Biol Sci*. 2017;372(1718):20160192.
13. Tomlinson CL, Stowe R, Patel S, Rick C, Gray R, Clarke CE. Systematic review of levodopa dose equivalency reporting in Parkinson's disease. *Mov Disord*. 2010;25(15):2649-2653.
14. Dixon WJ, Yuen KK. Trimming and winsorization: A review. *Stat Hefte*. 1974;15(2):157-170.
15. Maxwell SE, Delaney HD, Kelley K. *Designing Experiments and Analyzing Data: A Model Comparison Perspective*. Routledge; 2017.
16. Yogev-Seligmann G, Hausdorff JM, Giladi N. The role of executive function and attention in gait. *Mov Disord Off J Mov Disord Soc*. 2008;23(3):329-342.
17. Pieruccini-Faria F, Ehgoetz Martens KA, Silveira CR, Jones JA, Almeida QJ. Side of basal ganglia degeneration influences freezing of gait in Parkinson's disease. *Behav Neurosci*. 2015;129(2):214.
